# Supplementary material for: Acute Effects of Neuromuscular Electrical Stimulation on Contralateral Plantar Flexor Neuromuscular Function
Source: Biology (Basel). 2022 Nov 12;11(11):1655. doi: 10.3390/biology11111655 (PMC9687927; doi:10.3390/biology11111655)
Supplement: Supplementary file 1 [file biology-11-01655-s001.zip › biology-1983016-supplementary.pdf]

Supplementary Table S1. Ipsilateral torque recorded for each condition when electrical stimulation delivered to the contralateral tibial nerve

		Condition			
		REST	VOL	CONV	WPHF
	N				
H_{max}	10	0 (0 to 0)[a]	10.4 (9.8 to 10.6)[b]	5.4 (4 to 6.7)[c]	7.1 (6 to 14.7)[b]
M_{max}	10	0 (0 to 0)[a]	10.3 (9.9 to 11.3)[b]	9.1 (7.1 to 12.9)[b]	15.0 (10.5 to 28.7)[c]
Superimposed doublet	22	8.1 (-0.4 to 18.4)[a]	10.5 (10 to 11.5)[a]	15.5 (7.7 to 19.8)[a]	15.4 (11.2 to 30.8)[b]
M_{sup}	10	20.2 (9.8 to 33.3)[a]	10.9 (10.3 to 11.8)[a]	11 (6.4 to 31.1)[a]	26.1 (16.8 to 65.8) [b]
Potentiated doublet	22	0.6 (0 to 2.6)[a]	10.6 (9.3 to 12.3)[b]	12.8 (9.7 to 18.4)[b]	14.7 (6 to 37.9)[b]

Values are median (25th to 75th percentile) and expressed as % of ipsilateral MVC torque. Conditions that do not share a letter are significantly different from one another for that stimulation parameter ($P < 0.05$). H_{max} = stimulation to evoke a maximal H-reflex, M_{max} = stimulation to evoke a maximal M-wave. M_{sup} = stimulation at the intensity of M_{max} delivered during the maximal voluntary contraction. The number of participants is denoted by N. Due to problems with EMG data acquisition two subjects had to be excluded from these analyses.
